# Strategies for Safer Cefepime Use to Prevent Neurotoxicity Using the Electronic Health Record

**DOI:** 10.1097/CCE.0000000000001380

**Published:** 2026-02-11

**Authors:** Sahar F. Zafar, Hemi Park, Alyssa R. Letourneau, Alex E. Rock, M. Brandon Westover, Shibani S. Mukerji

**Affiliations:** 1 Department of Neurology, Massachusetts General Hospital, Harvard Medical School, Boston, MA.; 2 Harvard Medical School, Boston, MA.; 3 Vaccine and Immunotherapy Center, Division of Infectious Diseases, Massachusetts General Hospital, Boston, MA.; 4 Department of Medicine, Division of Infectious Diseases, Massachusetts General Hospital, Harvard Medical School, Boston, MA.; 5 Department of Pharmacy, Massachusetts General Hospital, Harvard Medical School, Boston, MA.; 6 Department of Neurology, Beth Israel Deaconess Medical Center, Boston, MA.

**Keywords:** antimicrobial stewardship, cefepime, delirium, electronic health record, neurotoxicity

## Abstract

Cefepime, a cornerstone antibiotic in critical care, is associated with underrecognized cefepime-induced neurotoxicity (CIN), particularly in patients 65 years old and older. The true incidence is unknown due to inconsistent monitoring and a lack of diagnostic criteria. The recent Antibiotic Choice on Renal Outcomes (ACORN) trial underscored CIN’s clinical significance, finding that cefepime recipients experienced 21% fewer delirium- and coma-free days than those on piperacillin-tazobactam. Current guidelines lack active surveillance recommendations, leading to delayed diagnosis and intervention. We propose three informatics-based strategies to address these challenges: 1) electronic health record (EHR)-integrated datasets utilizing machine learning and natural language processing to identify CIN at scale, 2) automated electroencephalogram tools to provide real-time alerts to clinicians, and 3) dynamic risk scores that continuously update from EHR data to guide prescribing. Implementing these safeguards to optimize CIN prevention, which may be relevant for other antibiotics with neurotoxicity risk, can improve neurologic outcomes and patient safety in critically ill populations.

## BACKGROUND

Cefepime, a fourth-generation cephalosporin, is commonly used in the treatment of hospital-acquired infections due to its broad-spectrum antimicrobial coverage and its ability to achieve therapeutic concentrations in a variety of tissue compartments (1). Use of third- and fourth-generation cephalosporins, including cefepime, has increased over past decades (2). Recent global data show that cephalosporin consumption rose across all income groups between 2016 and 2023, with particularly high rates in low- and middle-income countries, where usage averaged 4.9 defined daily doses of a cephalosporin per 1000 people per day. In contrast, fluoroquinolone use has declined significantly in high-income countries, largely due to regulatory warnings about disabling and potentially permanent side effects (3). Today, these cephalosporins are the most frequently prescribed antibiotics for patients 65 years old and older (4). During the COVID-19 pandemic, cefepime use increased significantly in U.S. hospitals, particularly among critically ill patients and in facilities with the highest COVID-19 burden, reflecting its role in empiric treatment of suspected bacterial co-infections (5). While other broad-spectrum antibiotics have also been associated with neurotoxicity, this commentary focuses on cefepime-induced neurotoxicity (CIN).

Although generally considered safe, cefepime was the subject of a 2012 U.S. Food and Drug Administration warning for nonconvulsive status epilepticus (NCSE) associated with inappropriate renal dosing (6,7). Notably, cefepime binds the γ‑aminobutyric acid type A (GABA_A_) receptor as a competitive antagonist in a concentration-dependent manner inhibiting GABA-induced electrical activity. This is thought to be a key mechanism for its neurotoxicity. Notably, CIN may also present as acute encephalopathy, defined as a rapidly developing pathobiological brain process presenting clinically as delirium (an acute disorder of attention and awareness with diagnostic features defined by systems such as the 5th edition of the diagnostic and statistical manual ) or coma (a clinical state of severely depressed responsiveness) (8). CIN is likely underrecognized due to multiple factors, with a key limitation being the lack of validated clinical diagnostic criteria. Notably, no established definitions of CIN currently incorporate electroencephalography findings as a diagnostic requirement (7,9–12). Cefepime levels vary widely between patients, increasing the risk of neurotoxicity, particularly in patients over 65 years old or with multiple comorbidities, who are already vulnerable to hospital delirium.

Despite its clinical significance, the true incidence of CIN remains unclear. Reviews show that CIN most often affects older adults with a near-equal sex distribution (13, 14). While underlying renal dysfunction, high doses of cefepime, and advanced age are well-recognized risk factors, other contributing factors also include preexisting CNS disorders and previous brain injuries (7, 9, 15, 16). In this commentary, we outline the current clinical challenges in recognizing and managing CIN and propose informatics-based strategies utilizing electronic health record (EHR) data and automated tools to improve early identification and prevention.

## ANTIBIOTIC CHOICE AND NEUROTOXICITY RISK

The Antibiotic Choice on Renal Outcomes (ACORN) trial was a key randomized clinical trial completed in 2022 that directly compared cefepime to piperacillin-tazobactam in 2511 hospitalized adults (17). The primary objective was to determine whether the choice between cefepime and piperacillin-tazobactam affects the risk of acute kidney injury or death within 14 days, with neurotoxicity as a key secondary outcome. Although there was no significant difference in the primary endpoint between the two groups (odds ratio, 0.95; 95% CI, 0.80–1.13; *p* = 0.56), cefepime was associated with worse neurologic outcomes. Specifically, patients treated with cefepime experienced 21% fewer delirium- and coma-free days within 14 days, as assessed by Confusion Assessment Method for the ICU (CAM-ICU) and Richmond Agitation-Sedation Scale scores, compared with those receiving piperacillin-tazobactam (mean ± sd: 11.9 ± 4.6 vs. 12.2 ± 4.3 d). Notably, the risk of neurotoxicity was elevated in patients with renal dysfunction, although cases occurred even with appropriate renal dose adjustments.

Although the difference in delirium-free days between the two groups was statistically significant, a 0.3-day difference was considered by some to be clinically modest. Additional limitations of the study included its single-center design, which reduces generalizability, and the administration of cefepime as a 2 g IV push over 5 minutes every 8 hours, which may have amplified neurotoxic side effects (18, 19). Furthermore, the trial was nonblinded, which could bias CAM-ICU assessments. There was treatment crossover (17.2–18.8% of patients received at least one dose of the alternate drug), and most patients (69%) were receiving neurologic medications at baseline, which could influence delirium outcomes. Additionally, the low ICU enrollment (4.2–6.5% across groups) limits generalizability to critically ill populations. Nonetheless, the ACORN trial and subsequent unresolved debate underscore CIN as a clinically relevant complication, with early recognition and intervention shown to improve outcomes (11).

## CLINICAL CHALLENGES

The lack of standardized guidance complicates CIN management. Current U.S. Infectious Diseases (ID), Neurology, and Critical Care guidelines do not recommend active surveillance for CIN, leaving practitioners without a consistent framework for diagnosis and management. Reported CIN incidence varies widely, from 0.2% in single-center retrospective reviews to 7.5% in therapeutic drug monitoring (TDM) studies, and up to 23% among hospitalized patients with renal disease (**Table 1**) (7, 10, 20, 21,24–26). This variability likely stems from reliance on small, single-center, and retrospective studies, or data from ICU cohorts, and differences in definition. Further, differences in diagnostic criteria, levels of renal dysfunction, and the severity of comorbidities may contribute to the inconsistency (12, 27). Typically, patients requiring broad-spectrum antibiotics present with complex clinical histories, including severe infection, metabolic derangements, and multimorbidity. These confounders make it challenging to isolate CIN as the primary cause of neurologic dysfunction. Manual chart reviews, often used in these studies, are time- and labor-intensive, limiting the number of cases that can be realistically analyzed (28). Practice or institutional variability in identifying delirium in acutely ill patients, ordering electroencephalograms for encephalopathy, or setting alerts for redosing renally cleared medications, also complicates accurate incidence estimation.

**TABLE 1.** Selected Studies Characterizing Cefepime-Induced Neurotoxicity^a^

Study	Study Design and Population	CIN Definition	Evaluation	Incidence, *n*/*N* (%)	Electroencephalogram Findings	Observations
Suttels et al (20)	Retrospective single-center, Switzerland; hospitalized noncritically ill, TDM (*n* = 119)	Side effects and/or neurotoxicity, defined as decreased level of consciousness, delirium, cognitive disturbances, myoclonus, nonconvulsive status epilepticus, seizures, or hallucinations after ≥ 2	Neurologic symptoms ≥ 2 d reviewed using electronic patient records for side effects and/or neurotoxicity, causality assessed via WHO scale; trough levels	9/119 (7.5)^b^	Not reported	High cefepime concentrations despite appropriate dosing; routine TDM recommended
Boschung-Pasquier et al (21)	Retrospective single-center, Switzerland; hospitalized, TDM (*n* = 319)	New-onset neurologic symptoms occurring after at least three doses (typically > 48 hr) of cefepime with no other likely cause	Neurologic symptoms after three doses, causality assessed via WHO-Uppsala Monitoring Centre; trough levels; confounding meds; improvement after discontinuation	74/319 (23.2)	Not reported	No neurotoxicity < 7.7 mg/L; 50% probability ≥ 16 mg/L; 100% ≥ 38.1 mg/L
Appa et al (10)	Systematic review of case reports/series (*n* = 193)	Neurologic symptoms characterized by diminished level of consciousness, disorientation, agitation, myoclonus, or seizures	Neurologic symptoms	Not reported	Performed in 141/174 (81%): triphasic waves, epileptiform discharges	Under-recognition emphasized; early cessation recommended; electroencephalogram useful for NCSE
	Retrospective single-center, United States; hospitalized (*n* = 5)	Suspected neurotoxicity cases identified via electronic safety networks and antimicrobial stewardship records	Neurologic symptoms, causality assessed via Naranjo Adverse Drug Reaction Probability Scale	5/2403 courses (0.21)	Performed in 2/5 (40%): triphasic waves, epileptiform discharges	Similar observations as literature; emphasizes under-recognition
Lamoth et al (22)	Retrospective single-center, Switzerland; hospitalized febrile neutropenic, TDM (*n* = 30)	Consistent neurologic signs or symptoms according to National Cancer Institute classification (grade II or III)	Neurologic symptoms ≥ 2 d; improvement 2 d after dose reduction	6/30 (20)	Not reported	50% CIN probability at trough ≥ 22 mg/L
Fugate et al (23)	Retrospective single-center, United States; ICU (*n* = 100)	Three-tiered criteria with: 1) neurologic symptoms such as encephalopathy, decreased alertness, myoclonus, or seizures; 2) clear temporal relationship with cefepime; and 3) no alternative cause	Neurologic symptoms, causality assessed using modified Delphi	15/100 (15)	Performed in 9/15 (60%); diffuse slowing, triphasic waves, multifocal sharp waves, NCSE	Mostly due to inappropriate renal dosing; some occurred despite correct dosing
Jeon et al (24)	Retrospective single-center, Korea; hospitalized first-time cefepime users (*n* = 1793)	Four-tiered criteria with: 1) altered consciousness or other specific neurologic symptoms after cefepime, 2) with no alternative cause including delirium or metabolic reasons, 3) recovery within a few days after cefepime discontinuation and full recovery to premorbid state, and 4) confirmed by physician	Altered consciousness during use; recovery after discontinuation; creatinine and eGFR	44/1793 (2.5)	Performed in 30/44 (68%): generalized periodic discharges with triphasic morphology, diffuse slowing, NCSE	Associated with low eGFR; delayed recovery common (> 7 d in > 50%)
Chatellier et al (25)	Retrospective case series, France; ICU (*n* = 5)	Neurologic symptom of seizure and coma with renal failure on cefepime	Neurologic symptoms, causality assessed via clinical judgment	Five cases in 2 yr (incidence not calculated)	Performed in all: diffuse slowing, sharp waves, paroxysmal bursts	Occurred exclusively in acute renal failure; hemodialysis led to recovery in 4/5 (80%)

CIN = cefepime-induced neurotoxicity, eGFR = estimated glomerular filtration rate, NCSE = nonconvulsive status epilepticus, TDM = therapeutic drug monitoring, WHO = World Health Organization.

a This table highlights selected studies and is not intended to be a comprehensive review of all published data on CIN.

b Suspected neurotoxicity (three probable cases, six possible cases).

TDM for cefepime is one proposed strategy for mitigating CIN risk, given that elevated plasma concentrations are strongly associated with neurotoxicity (20, 21). Plasma cefepime trough concentrations can be highly variable in critically ill individuals and some institutions have implemented TDM (21). However, TDM for cefepime is not routinely available in all clinical settings and is a resource limitation. Additionally, if TDM send-out testing is required, delayed turnaround times will reduce provider ability to make real-time adjustments in acute care settings.

The identification and management of CIN are challenging, especially regarding electroencephalogram use to detect subclinical seizures or periodic discharges, particularly when differentiating CIN and sepsis-associated encephalopathy. Generalized periodic discharges (GPDs) are common in patients receiving cefepime, often showing high-voltage morphology at a frequency of 2–3 Hz, sometimes referred to as “triphasic waves” (TWs). It is important to note that these patterns are not specific to CIN and can be observed in various metabolic conditions. The institutional variability in electroencephalogram interpretation and lack of universal American Clinical Neurophysiology Society terminology further complicates diagnosis.

There is debate about managing these findings with anti-seizure medications (ASMs) (12). Although GPDs do not always meet criteria for NCSE, empirical ASM trials may be beneficial in certain cases; one study found that 40% of patients with TW improved with ASM (29). There is ongoing debate on whether GPDs, particularly those occurring along the ictal-interictal continuum, warrant ASM therapy, or whether, in cases associated with toxic-metabolic derangements such as cefepime neurotoxicity, dose adjustment or discontinuation of cefepime alone may be sufficient (30–32). As with the epidemiology, further investigation is needed to better define the electroencephalogram findings and determine appropriate management in this context.

## AREAS FOR RESEARCH ADVANCES

Cefepime will likely remain a cornerstone of therapy in hospitalized patients, particularly those in the ICUs, for the near future (33, 34). While potential mitigation strategies, such as dose optimization, more frequent lower dosing, or extended infusion times tailored to infection type, have been proposed (15, 26), these approaches would be challenging to implement and are unlikely to be evaluated in randomized controlled trials. Importantly, the lack of standardized CIN diagnostic criteria complicates the interpretation of CIN research and the synthesis of proactive risk assessment tools. The lack of such tools means that interventions are largely reactive.

Ultimately, the development of approaches for early identification and prevention of CIN could meaningfully improve care, particularly in cases complicated by multiple confounding factors for encephalopathy. This also represents an important area of collaboration and research between neurology and antimicrobial stewardship efforts. While the proposed strategies are designed for CIN, they could inform broader neurologic stewardship efforts for other antibiotics. Several areas for infrastructure growth are considered potentially valuable for inpatient teams:

1) Development of institutional EHR datasets that integrate detailed clinical, laboratory, medication, TDM (where available), and electroencephalogram data at scale for the study of CIN. By identifying consecutive adult patients who received cefepime and applying natural language processing or large language model-based phenotyping tools to identify challenging neurologic conditions such as delirium, researchers can generate high-quality epidemiological evidence on the incidence, risk factors, and outcomes of CIN within hospital systems. We have developed several publicly available machine learning phenotyping tools through the Brain Data Science Portal (bdsp.io) to support large-scale phenotyping from clinical notes and welcome collaborations. These tools can facilitate the creation of comprehensive datasets to better characterize the clinical spectrum of CIN, inform clinical guidelines, and improve early detection to enhance patient safety.
2) Design of automated algorithms capable of identifying classic electroencephalogram patterns associated with CIN, such as GPDs, is a potential pragmatic next step. Several laboratories have expertise in automated electroencephalogram pattern recognition and are poised to demonstrate that such tools are both feasible and implementable in healthcare systems with electroencephalogram capabilities. By integrating electroencephalogram algorithms into clinical workflows, the aim would be to provide rapid, scalable alerts to clinicians, facilitating timely diagnosis and intervention. To support this effort, multicenter, real-world data from centers with electroencephalogram monitoring, especially where baseline electroencephalograms were available prior to cefepime initiation, would be highly valuable. These data could inform the design of a prospective study to evaluate neurotoxicity in cefepime-exposed patients. One approach is to pilot a protocol in which continuous electroencephalogram is co-ordered with cefepime in high-risk, critically ill patients. This could yield key insights into CIN-specific electroencephalogram changes and help validate automated detection algorithms. Although resource-intensive, logistically challenging and biased to centers with access to continuous electroencephalogram, such prospective studies can support refining algorithm development and optimizing electroencephalogram pattern recognition. This is particularly important if guidelines begin to recommend electroencephalogram monitoring for patients exposed to cefepime, as automated analysis will make such recommendations feasible on a large scale.
3) Develop and validate dynamic, point-based CIN risk scores, combining key clinical metadata, including renal function, age, and comorbidities, with electroencephalogram variables, to provide local clinicians with real-time, EHR-integrated early warning systems. A recent study developed a prediction score, but its generalizability was constrained by restrictive exclusion criteria, such as alcohol or substance use, history of epilepsy, dementia, or admission for CNS infection, and reliance on static risk factors like age, pre-cefepime renal function, and premorbid comorbidities (28). A dynamic risk score, continuously updating based on patient-derived information from the EHR, supports safer cefepime use while avoiding unnecessary empiric switches to alternative antibiotics. This is especially crucial in the current landscape, where options for treating multidrug-resistant infections are increasingly limited.

It is important to acknowledge that the proposed strategies depend on access to resources, including electroencephalogram monitoring and informatics infrastructure, which are not universally available. This inequity presents a barrier to optimizing CIN management using the approaches described above. At a minimum, establishing clear guidance across clinical practice guidelines and promoting the implementation of safer cefepime prescribing practices could lead to meaningful improvements in patient care.

## CONCLUSIONS

Cefepime remains a cornerstone of inpatient antibiotic therapy. Given the continued high usage of cefepime, its potential for neurotoxicity, and a rising proportion of individuals 65 and older in the United States, it is prudent to develop proactive risk management strategies and evidence-based guidelines for monitoring and intervention. Advances in technology, including large-scale data integration and automated analysis tools, now make it feasible to implement these safeguards against CIN and ensure patient safety in real-world clinical practice.
